# Exploiting the Capture Effect to Enhance RACH Performance in Cellular-Based M2M Communications

**DOI:** 10.3390/s17102169

**Published:** 2017-09-21

**Authors:** Jonghun Kim, Jaiyong Lee

**Affiliations:** Ubiquitous Network Laboratory, School of Electrical and Electronic Engineering, Yonsei university, 50 Yonsei-ro, Seodaemun-Gu, Seoul 03722, Korea; roro7773@yonsei.ac.kr

**Keywords:** M2M communications, machine-type communications (MTC), Internet of Things (IoT), cellular network, random access channel (RACH), congestion

## Abstract

Cellular-based machine-to-machine (M2M) communication is expected to facilitate services for the Internet of Things (IoT). However, because cellular networks are designed for human users, they have some limitations. Random access channel (RACH) congestion caused by massive access from M2M devices is one of the biggest factors hindering cellular-based M2M services because the RACH congestion causes random access (RA) throughput degradation and connection failures to the devices. In this paper, we show the possibility exploiting the capture effects, which have been known to have a positive impact on the wireless network system, on RA procedure for improving the RA performance of M2M devices. For this purpose, we analyze an RA procedure using a capture model. Through this analysis, we examine the effects of capture on RA performance and propose an Msg3 power-ramping (Msg3 PR) scheme to increase the capture probability (thereby increasing the RA success probability) even when severe RACH congestion problem occurs. The proposed analysis models are validated using simulations. The results show that the proposed scheme, with proper parameters, further improves the RA throughput and reduces the connection failure probability, by slightly increasing the energy consumption. Finally, we demonstrate the effects of coexistence with other RA-related schemes through simulation results.

## 1. Introduction

Supporting Internet of Things (IoT) services is emerging as one of the most important problems facing communication systems [1,2]. There are many applications, such as public safety, e-health, fleet management, and smart metering [1], that will use machine-to-machine (M2M) communication technology, which ensures connectivity to the devices without human interaction

Cellular networks are expected to play a major role in enabling M2M communications because they can provide seamless coverage over a large area, support mobility, and offer security. However, there are several problems with enabling M2M communications over cellular networks because these networks are optimized for human-to-human (H2H) communications and the characteristics of M2M communications are different from those of conventional H2H communications. For example, M2M communications are triggered by events, time-controlled, involve the transmission of small amounts of data, and are energy-limited [3]. The 3rd Generation Partnership Project (3GPP) has studied the issues facing M2M services in cellular networks. In [1], the 3GPP studied potential system improvements for M2M communications, the authors organized the architectural considerations and defined the expected problems for M2M communications. One of the most critical problems is congestion due to the massive access by M2M devices. The control plane at the radio-access-network (RAN) level suffers during massive access by M2M devices due to their characteristics. M2M devices try to establish a connection with an evolved node B (eNB) when they have data to transmit, causing a severe random access channel (RACH) congestion problem. The 3GPP suggested some solutions [1], including an access class barring (ACB) scheme, a separate RACH resource-allocation scheme, a dynamic RACH resource-allocation scheme, and an M2M-specific back-off scheme.

Because RACH congestion due to massive access causes the degradation of RA throughput, increasing the RA throughput has become a major research target. The ACB method efficiently solves the RA throughput problem by controlling the access probabilities of separate access classes. In [4], the authors suggested dynamic-ACB (D-ACB) to not only overcome the RACH congestion problem due to bursts of M2M traffic but also to reduce the total service time. The D-ACB determines the accurate ACB factor without backlog information. In [5], a cooperative ACB (C-ACB) scheme was proposed to reduce the congestion in multi-cell cases. In this study, the ACB factors of each eNB were used for the eNB selection by M2M devices. Furthermore, the ACB factors were jointly determined among the neighboring eNBs. The access load could be distributed using the cooperative method. In [6], timing-advance (TA) information was used to reduce the RA collision probability because the propagation delay between a stationary M2M device and the eNB is a constant. Hence, the authors assumed that the TA values in a random access response (Msg2) could be used as an identifier; therefore, some preamble collisions could be solved. Based on this, they proposed a TA-based ACB scheme for increasing RA throughput. In [7], an ACB scheme for delay-sensitive devices is proposed. The authors consider the scenario where delay-sensitive and delay-tolerant services coexist. In addition, a priority-based ACB scheme adjusts dynamically the access probability and the number of preambles to satisfy the requirements of delay-sensitive services. In addition, delay-tolerant services can also be highly utilized based-on preamble allocation.

A preamble-separation method has also been proposed to increase the RA throughput. In [8], two preamble-allocation methods for M2M/H2H services were studied. One method split the preambles into disjointed sets for M2M and H2H separately, while the other split them into an H2H-only set and an H2H/M2M-hybrid set. The authors modeled these methods and analyzed them in terms of throughput. In [9], the authors analyzed the non-overlapping preamble separation, proposing the use of a load-adaptive throughput-maximizing preamble-allocation procedure, which automatically adjusted the preamble allocation to the priority classes, based on the access load. Both studies showed that the preamble–separation scheme increased the RA throughput, especially when the access load was heavy.

The energy consumption of M2M devices is also a very important issue because most M2M devices are vulnerable to energy consumption. Therefore, many studies have been conducted on the energy consumption of the RA process. In [10], further improvement of traffic scattering for the group paging (FI-TSFGP) scheme was proposed to achieve high channel-access probability for M2M services. They also provided analysis of the scheme in terms of access latency and power consumption. Consequently, they argued that the FI-TSFGP scheme led to the reduction of both the channel access latency and the power consumption. In [11], group-based access control was studied in terms of the energy consumption of M2M devices. The authors introduced a coordinator to reduce massive access. In addition, they proposed a joint massive access control and resource allocation scheme that minimized total energy consumption.

Analyzing the RA procedure is fundamental to understanding the RACH congestion problem and to providing the proper solutions. Various studies have analyzed an RA procedure to solve the RACH problem. These studies have mostly focused on the well-known multi-slotted Advocates of Linux Open-source Hawaii Association (ALOHA) model. The RA throughput, access delay, efficiency, and energy consumption of the RA procedure have been well analyzed using the multi-slotted ALOHA model [4,5,6,12,13,14]. However, these studies used the classical collision model to interpret RA nature, which assumes that no signal is decoded when multiple nodes transmit simultaneously. This model allows for simple analysis but does not yield accurate results due to the characteristics of wireless communications. Other studies have used the slotted-ALOHA system in a capture model [15,16]. This model assumed that a certain signal could be decoded when the received power of a specific signal was larger than a predefined threshold. Studies exploiting this model have provided more realistic results.

In this paper, we provide novel solution to resolve the RACH congestion problem due to massive access of M2M devices by exploiting the capture effect: Msg3 power ramping (PR) scheme. The main contributions of this paper are summarized as follows:

1. We formulated analysis models of the RA procedure considering the capture effect. Previous studies [4,5,6,12,13,14] adopted the collision model to analyze the RA procedure and assumed that the RA performance degradation due to the collisions of the preambles (Msg1) could not be prevented. However, we provide an RA analysis model that considers the capture effect. Therefore, we consider RA performance improvements due to the capture effect of the physical layer, even if the collision occurs. We provide an RA performance analysis model, in terms of the RA throughput, the connection failure probability, and the average energy consumption of the devices.
2. Exploiting the capture effect to improve the RA performances, we propose the Msg3 PR scheme. This scheme causes a difference in the received power of messages at the eNB, so that the capture effect occurs frequently. In addition, it is easy to apply because the proposed scheme adopts the power ramping scheme that has already been used in the cellular system. Moreover, by applying this new technique, a device that has performed many retransmissions would have a higher RA success probability; thus, the connection failure probability could be reduced.
3. In the simulation, we demonstrate the accuracy of proposed analysis and the advantages of the Msg3 PR scheme. In addition, the effects of various network parameters on the performance of the technology are demonstrated. Moreover, we discuss the beneficial points of the proposed scheme when used with other RA-related schemes.

The rest of the paper is organized as follows. In Section 2, we introduce our system model. The motivation of this study and an introduction to the Msg3 PR scheme are presented in Section 3. In Section 4, we derive the analysis models for the RA procedure, considering the capture effect, and provide the performance of the proposed scheme, in terms of several performance metrics. In Section 5, the simulation results demonstrate the performance of the Msg3 PR scheme and the accuracy of the proposed analysis. Finally, Section 6 presents the conclusions.

## 2. System Model

### 2.1. Random Access Procedure

An M2M device in the radio-resource-control (RRC) idle mode performs an RA procedure to set up a connection with an eNB before data transmission. There are two types of RA procedures: contention-free and contention-based. In this study, we focus on the contention-based RA procedure. Before beginning the RA, the device listens to the system information block (SIB) messages broadcasted periodically by the eNB. The SIB messages contain information about the RA, such as the physical random access channel (PRACH) configuration index and the frequency offset, which inform about what resource blocks are reserved for the subsequent RA. Furthermore, information about which preambles are reserved for groups and about power control is included in the SIB messages. The contention-based RA procedure includes four steps messages exchanging [17]; the RA procedure modeled in this study is as follows:

1. Msg1. Preamble transmission
  The RA procedure is initiated by the transmission of a preamble that is randomly chosen from preamble set *V* at a predetermined RA slot. As a result, there are three cases: singleton preamble, collided preambleand empty preamble. Singleton preamble is chosen by exactly one device, the collided preamble preamble is chosen by multiple devices, and the empty preamble is chosen by none of the devices. An eNB can only detect whether a specific preamble is transmitted, but cannot determine whether it is a collided preamble or a singleton preamble [17,18]. Devices that send Msg1 set the random-access-response (RAR) window.
2. Msg2. RAR
  When the eNB detects a preamble transmission, it sends an Msg2 for the corresponding preambles through the physical-downlink control channel (PDCCH). This Msg2 contains the radio-network temporary identifier (RA-RNTI), the timing-advance (TA) information, and the uplink grant. The RA-RNTI identifies the corresponding preamble, the TA information is used to adjust the synchronization of the corresponding device, and the uplink grant is used to schedule the transmission of Msg3. Because the eNB cannot distinguish between singleton and collided preambles, it sends Msg2 for either preamble. Therefore, multiple devices that select the same preamble during Msg1 transmission receive the same Msg2, which only indicates the corresponding preamble. If a device does not receive an Msg2 within the RAR window, it realizes that the current RA has failed.
3. Msg3. RRC-connection request
  After sending Msg1, the device observes the PDCCH for the RAR window. If it finds the corresponding Msg2, it sends the Msg3, containing its identifier, by using a physical-uplink shared channel (PUSCH) informed by the uplink grant in the Msg2. Devices that send Msg3 set the contention–resolution time. Devices that choose the same preamble (i.e., collided preamble) also send an Msg3 containing their own identifier by using the same uplink grant, and, therefore, an Msg3 collision can occur.
4. Msg4. RRC-connection setup
  The eNB transmits the Msg4 using the PDCCH to inform the device that an RRC connection has been set up. Because the Msg4 contains the device identifier received from successfully received Msg3, an RA collision can be resolved if a specific Msg3 is decoded by capture effect.

If any of the four steps fails, an RA failure occurs. In this study, we assume that RA failure only occurs when the devices fail to receive an Msg 4. If a device does not receive an Msg 4 within the contention-resolution time, it performs a random back-off and re-attempts the RA, if the trial number does not exceed the maximum trial number, $N_{max}$. If the trial number exceeds $N_{max}$, a *connection failure* occurs. Note that the trial number can be from 1 to $N_{max}$. If the RA procedure is successful within $N_{max}$ RA trials, the device successfully connects to the eNB. We do not consider the hybrid automatic repeat request (HARQ) procedure for Msg3 or Msg4, nor do we consider the PDCCH resource constraint.

### 2.2. Power Capture Effect

Due to the characteristics of wireless communications, a specific signal can be decoded when multiple signals are received at different powers, causing the capture effect. This phenomenon occurs when the strongest signal power received from a specific device is sufficiently large. In other words, the capture effect occurs when the signal-to-interference ratio (SIR) (in [16], the signal-to-interference-plus-noise-ratio (SINR) is used. However, we use SIR to model the capture effect as [15] for mathematical tractability) of the strongest signal is larger than a specific threshold [15,16]. If $P_{i}$ denotes the signal power received from device *i* at the eNB, the probability that the signal from device *i* is captured, i.e., $P_{pc,i}$, is defined as follows:

(1) $$P_{pc,i}=P\left(P_{i}>\beta \sum_{j\neq i}P_{j}\right),$$

where β is *the capture-threshold*.

### 2.3. Uplink Power Control

In [19], the Msg1 transmission adopted *a power-ramping scheme* to increase the probability of preamble detection, which was not considered in this study. When a device re-attempts the RA procedure after back-off, it increases its Msg1 transmission power through a power-ramping. Let $P_{msg1}$ denote the Msg1 transmission power, and $P_{msg1}$ can be defined as follows [10,17]:

(2) $$P_{msg1}=\min\left(P_{Cmax},PRTP+PL\right),$$

where $P_{Cmax}$[dBm] is the maximum transmission power, PL[dB] is the path loss; and PRTP[dBm] is *the preamble received target power*, which is defined as follows:

(3) PRTP=PIRT+delta+(r−1)PRS.

Here, PIRT is *the power initial received target*, delta is *the offset value* depending upon the preamble format, and *r* is *the ramping number* that is associated with the trial number of the device. Using open- and closed-loop power control [19], a device can estimate the PL and, therefore, can adjust its transmission power to meet the target received power at the eNB. In this study, we assume that the power control is performed perfectly, such that the received signal power depends only upon the ramping number.

The Msg3 transmission power follows the PUSCH power control policy [18]. The Msg3 transmission power is affected by the modulation and coding scheme (MCS), resource blocks, and other factors. For the case of M2M communications, the data required is quite small, and the MCS is also low due to the low complexity. Hence, it is reasonable that the transmission power of Msg3 is identical for M2M devices. Consequently, we assume, for simplicity, that

(4) $$P_{msg3}=\min\left(P_{Cmax},P_{target}+PL\right),$$

where $P_{target}=10\log M+P_{0}+delta_{MCS}+f\left(i\right)$ is assumed to be a constant value. Here, *M* is the number of assigned resource blocks, $delta_{MCS}$ is the change in value related to MCS, and the other values are defined by the high-layer.

## 3. Motivation and Proposed Scheme

The RACH congestion problem can be fatal for a network system and devices. As described in the RA procedure in Section 2.1, a collision occurs when multiple devices select the same preamble and send their Msg1s. Because this Msg1 collision cannot be detected by the eNB during the Msg1 reception, these devices suffer from an Msg3 collision that also wastes the PUSCH resources. Moreover, the congestion problem gets worse due to the retransmission by the RA-failed devices and can even cause a connection failure. In summary, the RACH congestion problem causes (1) degradation of the RACH throughput, (2) increased probability of connection failures, and (3) wasting of the uplink resources (PUSCH). Exploiting the capture effect positively affects the network system [15,16]. Intuitively, the RACH congestion problem can also benefit from exploiting the capture effect. Even though collisions occur, if an Msg3 of a specific device is captured, the eNB can send an Msg4 to the device, and, therefore, the device would successfully connect. The received signal powers at the eNB are differentiated to exploit the capture effect. However, as seen in Equation (4), there is no difference between the target powers of the received signals when perfect power control for M2M devices is performed. Hence, we propose adopting a power ramping scheme for Msg3 transmission to exploit the capture effect. The proposed scheme leads the device to transmit at higher power as more retransmissions occur. There are some advantages using the power ramping scheme: first, it is easy to implement because it has been adopted for Msg1 transmission; and, second, the more retransmitted devices there are, the higher the probability of being captured, reducing the probability of connection failure. Because the proposed scheme uses a power-ramping scheme, the Msg3 transmission power can be reformulated as follows:

(5) $$P_{msg3}=\min\left(P_{Cmax},P_{target}+PL+\left(r-1\right)PRS\right).$$

As a result, the received power can be differentiated based on the ramping number (*r*) of the device.

## 4. Analysis Model

Intuitively, the Msg3 PR will have a positive effect on the probability of RA success because it helps the eNB capture the Msg3, even in a collision situation. However, it is necessary to analyze how the proposed scheme is affected by the network parameters and to analyze negative aspects of the scheme, such as the increased energy consumption of the devices. In this section, we propose an RA procedure analysis model that considers the capture effect in the Msg3 reception. For mathematical tractability, we assume that network is in a steady-state (in [4,10,14], a heavily congested network reaches a steady-state. In other words, the number of arrivals of retransmitted devices is much greater than the number of new arrivals. When the network is in a steady state, the network states (ex. the number of RA arrivals, the distribution of the trial numbers in the network, the number of RA successes at each RA procedure, etc.) are merely constant). We provide an analytic model for three performance metrics: RA throughput, connection failure probability, and average energy consumption. The RA throughput and connection failure probability demonstrate the positive effect of the proposed Msg3 PR scheme, while the average energy consumption is a potential negative effect of the proposed scheme. Table 1 summarizes the notations used in this analysis.

### 4.1. System Model

We consider a single cell with radius $X^{cell}$. Let N and *N* denote the set of devices sending Msg1 to participate in RA at the same RA slot, and the size of N, respectively. Devices in N are uniformly distributed in a cell, hence, $X_{u}=x$ which denotes the distance between the eNB and *u*, follows a uniform distribution. A device u∈N, which failed the RA procedure (t−1) times and participates in the $t^{th}$ RA at this RA slot has a trial number $T_{u}=t$. Similarly, $R_{u}=r$ denotes the ramping number of a device defined by Equation (5) at the current RA slot. As seen in Equation (5), the Msg3 transmission power of *u* is upper-bounded by $P_{Cmax}$ as $P_{target}+\left(r-1\right)PRS+PL\leq P_{Cmax}$. Note that PL is related to *x*. For simplicity, PL can be defined using the log-distance path loss model without fading as $PL\left(x\right)={PL}_{0}+10\alpha \log\left(\frac{x}{x_{0}}\right),$ where ${PL}_{0}$ and $x_{0}$ are the reference-path loss and distance, respectively. From the upper-bound condition and the path-loss model, the upper-bound of the ramping number of the device located at a distance *x* from the eNB is as follows:

(6) $$r\leq \max\left(1,\left\lfloor \frac{P_{Cmax}-PL\left(x\right)-P_{target}}{PRS}+1\right\rfloor \right)=K_{max}\left(x\right).$$

**Definition** **1.** *$K_{max}\left(x\right)$ is the maximum allowable ramping number of a device located at a distance x from the eNB. Then, the ramping number of u, $R_{u}$, can be defined as follows:*

(7) $$R_{u}=\min\left(K_{max}\left(x_{u}\right),T_{u}\right).$$

**Figure 1:**
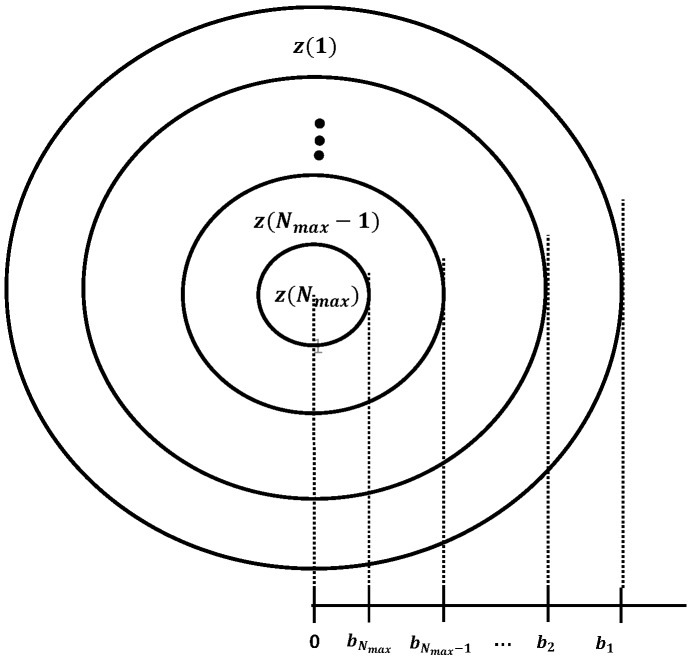
Definition of ’zone’.

However, Equation (7) is hard to deal with. Hence, we introduce the concept of *zones* for tractability as shown in Figure 1.

**Definition** **2.** Zone k is the area in which devices with $K_{max}=k$ are located. In addition, $Z_{u}$ is the zone in which device u belongs. Thus, $Z_{u}=k$ means that a device u is in zone k (also, u can only increase its ramping number up to k). Therefore, $zone\;N_{max}$ is the closet to the eNB, whereas zone1 is the farthest from the eNB.

If the distribution of $X_{u}$ is known (i.e., the distribution of devices in a cell), the probability that device *u* belongs to *zone k*, $P(Z_{u}=k)$, is (see Figure 1)

(8) $$P\left(Z_{u}=k\right)=\int_{b_{k+1}}^{b_{k}}P\left(X_{u}=x\right)dx,\;k\in \left[1,N_{max}\right].$$

Note that $K_{max}\left(x\right)$ is a decreasing function for *x*. Therefore, there exists a boundary distance $b_{k}=K_{max}^{-1}\left(k\right),\;k=1,\ldots ,N_{max},$ where $b_{N_{max}+1}=0$.

**Definition** **3.** $E[N_{T=t}^{k}]$ is the expected number of devices having a trial number t that are located in zone k for $t\in [1,N_{max}]$ and $k\in [1,N_{max}]$. Then, $\sum_{k}\sum_{t}E\left[N_{T=t}^{k}\right]=N$ holds.

If $E[N_{T=t}^{k}]$ for ∀t,k are given, we can define the distribution of the ramping number.

**Definition** **4.** $P(R_{u}=r,Z_{u}=k\;|\;N)$ is the probability that a device has a ramping number r and is located in zone k; $E[N_{R=r}^{k}]$ is the expected number of devices that have a trial number r and are located in zone k when N devices participate in the RA for $r\in [1,N_{max}]$ and $k\in [1,N_{max}]$.

$E[N_{R=r}^{k}]$ can be derived from $E[N_{T=t}^{k}]$. Because the devices belonging to *zone k* can only increase their ramping numbers to *k*, $N_{R=r}^{k}=0$ for r>k. Hence, $E[N_{R=r}^{k}]$ can be defined using $E[N_{T=t}^{k}]$ as follows:

(9) $$\begin{array}{c} E\left[N_{R=r}^{k}\right]=\left\{\begin{array}{cc} E[N_{T=r}^{k}], & 1\leq r<k,\\ \sum_{t=k}^{N_{max}}E\left[N_{T=t}^{k}\right], & r=k,\;\;\;\;,\;r\in [1,N_{max}],\\ 0, & k<r\leq N_{\max}. \end{array}\right. \end{array}$$

Finally, the following two equations are derived.

(10) $$\begin{array}{cc} P(R_{u},Z_{u}=k|N) & =\frac{E[N_{R=r}^{k}]}{N}\\ P(R_{u}|N) & =\sum_{k=1}^{N_{max}}P\left(R_{u},Z_{u}=k|N\right)\\ & =\frac{E[n_{R=r}]}{N},\;r\in \left[1,N_{max}\right], \end{array}$$

where $P(R_{u}|N)$ is the distribution of the ramping number when the number of accesses is *N*.

### 4.2. RA Throughput

In this sub-section, we provide an analysis model for the RA throughput. First, we provide a one-shot model when *N* and $E[N_{T=t}^{k}]$ for ∀t,k are given. Then, we demonstrate a steady-state RA throughput using the one-shot model because $E[N_{T=t}^{k}]$ is affected by the RA success probability, as determined by the RA throughput.

#### 4.2.1. One-Shot RA Throughput Analysis

Devices in N send an Msg1 that is randomly selected from *V* preambles. Focusing on a certain device, u∈N with $T_{u}=t$ and $Z_{u}=k$, and, therefore, $R_{u}=r=min\left(t,k\right)$. Let $V_{u}$ denote the preamble selected by device *u*, and $M_{v}$ denote the number of devices that select preamble *v* among N excluding device *u*. Then, the probability that device *u* and *m* devices in set $N\backslash \left\{u\right\}$ select preamble *v* can be expressed as follows:

(11) P(Mv=m,Vu=v|N)=1VN−1m1Vm1−1VN−m−1,m∈[0,N−1],v∈[1,V].

Next, we derive the probability that device *u* succeeds in a RA (i.e., the Msg3 from *u* is successfully decoded by the eNB), which we denote as the event $S_{u}$. Let the random variable $P_{i}^{rx}$ denote the received signal power from device *i* at the eNB. Then, the event $\left(S_{u}\right)$ occurs, even if *u* collides with *m* devices, when $P_{u}^{rx}$ meets the capture condition defined in Equation (1). Hence, the conditional probability of ($S_{u}$) is

(12) $$P\left(S_{u}\;|\;N,M_{v}=m,V_{u}=v,P_{u}^{rx}=p\right)=P\left(\beta \sum_{i\in M_{v}}P_{i}^{rx}\leq p\;|\;N,m,v,P_{u}^{rx}=p\right).$$

Since we assume that power control with full compensation of path loss is performed perfectly, $P_{i}^{rx}$ is a function of the ramping number as in Equation (5). Therefore, the probability density function (PDF) of $P_{i}^{rx}$ follows the distribution of the ramping number $\left(R_{i}\right)$ of the RA-participating devices as Equation (10). In other words, the PDF of $P_{i}^{rx}$ is

(13) $$P\left(P_{i}^{rx}=P^{rx}\left(r\right)\;|\;N\right)=P\left(R_{i}=r|N\right).$$

Hence, the conditional probability in Equation (12) is redefined as $R_{u}$:

(14) $$\begin{array}{c} P\left(S_{u}|N,M_{v}=m,V_{u}=v,P_{u}^{rx}=P^{rx}\left(r\right)\right)\\ \;=P(S_{u}|N,M_{v}=m,V_{u}=v,R_{u}=r)\\ \;=P\left(\beta \sum_{i\in M_{v}}P_{i}^{rx}\leq P^{rx}\left(r\right)\;|\;N,m,R_{u}=r\right)\\ \;={CP}_{N,m,r}, \end{array}$$

where ${CP}_{N,m,r}$ is the Msg3 capture probability, which means the probability that device *u* whose ramping number is *r* is captured by the eNB when *m* devices among *N* devices collide with *u*. Note that ${CP}_{N,0,r}$ is the capture probability when *u* selects the singleton preamble, and therefore, ${CP}_{N,0,r}=1$ for any *N* and *r*. Because the PDF of $P_{i}^{rx}$ is defined, and is independent and identically distributed, *i.i.d.*, Equation (14) can be solved using *the m-fold convolution* of the PDF of $P_{i}^{rx}$ for given values of N,m, and *r* [16].

Then, the following conditional probability can be obtained from Equations (8), (10) and (14), and *the chain rule*:

(15) P(Su,Ru=r,Zu=k|N,Mv=m,Vu=v) =P(Su|N,Mv=m,Vu=v,Ru=r,Zu=k)P(Ru=r|N,Mv=m,Vu=v,Zu=k)12341234P(Zu=k|N,Mv=m,Vu=v) =CPN,m,rP(Ru=r|N,Zu=k)P(Zu=k),r∈[1,Nmax],k∈[1,Nmax].

Note that (a) $S_{u}$ is dependent of $R_{u}$, not $Z_{u}$, (b) $R_{u}$ is independent of $V_{u}$ and $M_{v}$, and (c) $Z_{u}$ is independent of $N,M_{v}$, and $V_{u}$, so Equation (15) holds.

From Equations (11) and (15), we have

(16) P(Su,Mv=m,Vu=v,Ru=r,Zu=k|N)P1p2p2p2p2p2p2p2p2p2p2p2p2p2p2p2p=P(Mv=m,Vu=v|N)P(Su,Ru=r,Zu=k|N,Mv=m,Vu=v)m∈[0,N−1],v∈[1,V],r∈[1,Nmax],k∈[1,Nmax].

Thus, the probability that an arbitrary device succeeds in RA when *N* devices participate in the RA is

(17) P(Su|N)=∑v=1V∑m=0N−1∑r=1Nmax∑k=1NmaxP(Su,Mv=m,Vu=v,Ru=r,Zu=k|N)=∑m=0N−1N−1m1Vm1−1VN−m−1∑r=1NmaxP(Su|N,Mv=m,Ru=r)123∑k=1NmaxP(Ru=r|N,Zu=k)P(Zu=k)=∑m=0N−1N−1m1Vm1−1VN−m−1∑r=1NmaxCPN,m,rP(Ru=r|N=N)=∑m=0N−1N−1m1Vm1−1VN−m−1CPN,m,

where ${CP}_{N,m}=\sum_{r=1}^{N_{max}}{CP}_{N,m,r}P\left(R_{u}=r|N\right),$ which denotes the capture probability of a device when it collides with *m* devices among *N* devices.

Consequently, we can obtain the one-shot RA throughput when *N* devices participate in RA as

(18) E[S|N]=NP(Su|N)=N∑m=0N−1N−1m1Vm1−1VN−m−1CPN,m.

#### 4.2.2. Steady-State RA Throughput Analysis Model

As seen in Equation (14), the capture probability, $CP_{N,m,r}$, is affected by the distribution of the ramping number $P(R_{u}|N)$, which is determined by $E[N_{T=t}^{k}]$ as in Equation (9). Therefore, $E[N_{T=t}^{k}]$ must first be obtained. Let us assume that the network is in a steady-state with a stable number of RA accesses *N* at each RA slot; then, with a slight abuse of notation, we can still represent *N* and $N_{T=t}^{k}$ as steady-state values. Then, the following condition holds.

**Figure 2:**
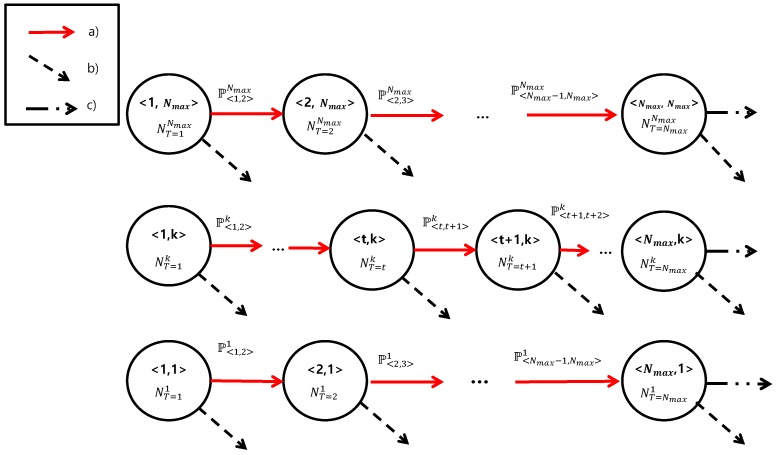
Markov chain model of the devices. State <t,k> indicates that the devices with the trial number $T_{u}=t$ and $Z_{u}=k$. Note that state transitions occur when (**a**) random acess (RA) failure, (**b**) RA success, and (**c**) connection failure occurs, respectively.

Note that $E\left[N_{T=1}\right]=\sum_{k}E\left[N_{T=1}^{k}\right]$ denotes the number of new arrivals. Now, we introduce a Markov chain model to determine the state of the devices, where state <t,k> presents those devices with a trial number *t* and are located in zonek. If we assume that the M2M devices have no mobility, a state transition only occurs when a device (a) fails in RA and retries (from <t,k> to <t+1,k>), (b) succeeds in RA, or (c) fails in its connection set up. Figure 2 presents this Markov chanin model. Then, $P_{<t,t+1>}^{k}$ can be derived as follows:

(19) $$\begin{array}{cc} P_{<t,t+1>}^{k} & =1-P(S_{u}|N,T_{u}=t,Z_{u}=k)\\ & =1-P(S_{u}|N,R_{u}=\min\left(t,k\right))\\ & \;\;\;t\in \left[1,N_{max}-1\right],\;k\in \left[1,N_{max}\right], \end{array}$$

where

(20) P(Su|N,Ru=r)=P(Su,Ru=r|N)P(Ru=r|N)=∑m=0N−1N−1m1Vm1−1VN−m−1CPN,m,r.

Finally, we can obtain the steady-state value $E[N_{T=t}^{k}]$ using balance equation property.

(21) $$\begin{array}{c} E\left[n_{T=t+1}^{k}\right]=\left\{\begin{array}{cc} P\left(Z_{u}=k\right)E\left[N_{T=1}\right] & ,\;t=1,\\ P_{<t,t+1>}^{k}E\left[n_{T=t}^{k}\right] & ,\;1<t\leq N_{\max}-1, \end{array}\right. \end{array}$$

(22) $$\sum_{t=1}^{N_{max}}\sum_{k=1}^{N_{max}}E\left[N_{T=t}^{k}\right]=N.$$

**Figure 3:**
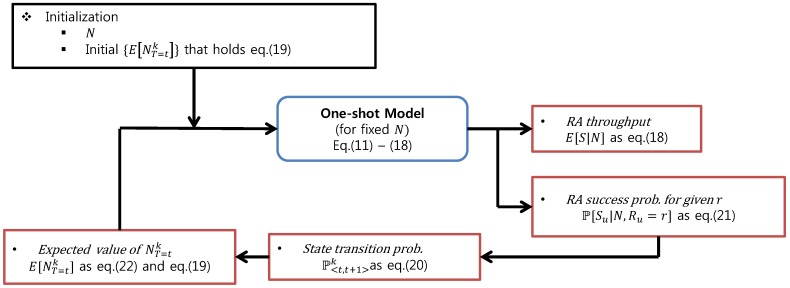
Summary for the iterative derivation of the steady state RA throughput.

Here, the first line of Equation (21) holds because the locations of devices are uniformly distributed. From Equations (21) and (22), $E[N_{T=t}^{k}],\forall t,k$ can be obtained. In summary, the steady-state RA throughput for fixed the number of accesses, *N*, can be obtained by the iterative procedure as in Figure 3.

### 4.3. Connection Failure Probability

Connection failure probability is defined as the probability that a device fails to acquire connectivity by failing the RA process $N_{max}$ times. Let $P_{CS,T=t|N,Z=k}$ and $P_{CF,T=t|N,Z=k}$ denote the conditional connection success and connection failure probabilities, respectively, of a device with a trial number *t* for given zonek and total arrival *N*, respectively. Therefore, $P_{CS,T=t|N,Z=k}$ is equal to the probability that the device fails at RA (t−1) times, and finally succeeds in the $t^{th}$ RA trial. Then,

(23) $$\begin{array}{c} P_{CS,T=t|N,Z=k}=\left\{\begin{array}{cc} P_{S|N,R=r}\prod_{i=1}^{t-1}\left(1-P_{S|N,R=i}\right), & \mathrm{if}t\leq k,\\ P_{S|N,R=k}{\left(1-P_{S|N,R=k}\right)}^{t-k}\prod_{i=1}^{k-1}\left(1-P_{S|N,R=i}\right), & \mathrm{if},\;t\;>\;k, \end{array}\right. \end{array}$$

where $P_{S|N,R=r}$ is the simple notation of $P(S_{u}|N,R_{u}=r)$, as defined in Equation (20). Then, the connection failure event is same as the complementary event of the connection success cases. Thus, $P_{CF|N,Z=k}$ can be derived as follows:

(24) $$\begin{array}{cc} P_{CF|N,Z=k} & =1-\sum_{t=1}^{N_{max}}P_{CS,T=t|N,Z=k}\\ & =1-\sum_{r=1}^{k}P_{S|N,R=r}\prod_{i=1}^{r-1}\left(1-P_{S|N,R=i}\right)-P_{S|N,R=k}\prod_{i=1}^{k-1}\left(1-P_{S|N,R=i}\right)\sum_{t=k+1}^{N_{max}}{\left(1-P_{S|N,R=k}\right)}^{t-k}. \end{array}$$

Finally, the connection failure probability, $P_{CF|N}$, can be derived using Equation (8) as follows:

(25) $$P_{CF|N}=\sum_{k=1}^{N_{max}}P_{CF|N,Z=k}P\left(Z_{u}=k\right).$$

### 4.4. Average Energy Consumption

#### 4.4.1. Energy Consumption Model

**Figure 4:**
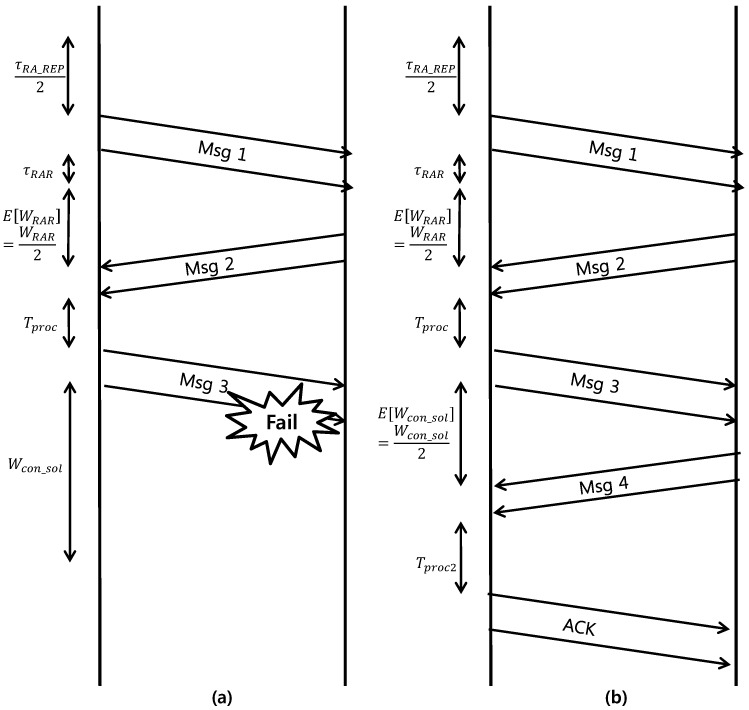
The power consumption model for (**a**) single failed RA event (**b**) single successful RA event.

An average energy consumption model for the devices needs to be defined to analyze the energy consumption of the proposed scheme. For the energy-consumption parameters, we have partially referenced the model used in [10]. In [10], the authors assumed that an RA collision is detected by the eNB during the Msg1 reception, whereas we assumed that a collision is detected when the eNB cannot decode Msg3. Therefore, we refer to and modify the energy consumption model defined in [10]. In this study, the description of how parameters are defined is omitted. See [10] for a detailed description of the parameters. Instead, we provide simplified description in Figure 4. Let $P_{1},P_{2}$, and $P_{3}$ denote the power consumption for waiting RA slot and performing back-off, receiving messages from the eNB, and transmission, respectively. Here, for analytic convenience, we assume that all transmission powers are equal, i.e., $P_{3}=P_{msg1}=P_{msg3}$ as in Equation (5). Then, the average energy consumption for failed RA and successful RA events can be modeled as follows.

1. The energy consumed during a failed RA event is equal to that consumed in the following steps: waiting for the first RA slots($\left(\tau_{RA\_REP}/2\right)P_{1}$), transmitting Msg1 ($P_{3}$), waiting for the RAR window ($\tau_{RAR}P_{1}$), wating for the Msg2 ($\left(W_{RAR}/2\right)P_{2}$), receiving the Msg2 ($\tau_{msg2}P_{2}$), processing time for Msg3 ($T_{proc}P_{2}$), transmitting Msg3 ($P_{3}$), and expiring the contention-resolution timer ($W_{con\_sol}P_{2}$).
2. The energy consumed during a successful RA event is equal to that consumed during the following steps. From transmitting Msg1 to receiving Msg2, the power consumed by the successful RA follows that consumed by the failed RA procedure and equals $\left(\left(\tau_{RA\_REP}/2\right)P_{1}+P_{3}+\tau_{RAR}P_{1}+\left(W_{RAR}/2\right)P_{2}+\tau_{msg2}P_{2}+T_{proc}P_{2}+P_{3}\right)$. In addition, the following steps are: for waiting Msg4 ($\left(W_{con\_sol}/2\right)P_{2}$), the power consumed when receiving Msg4 ($\tau_{msg4}P_{2}$), processing time for acknowledgement (ACK) ($T_{proc2}P_{2}$), and the power consumed by transmitting the ACK of Msg4 ($P_{3}$).

Note that 1/2 terms denote the average time values. Parameter values in the above model are described in Table 2.

#### 4.4.2. Average Energy Consumption

$\delta_{F}$ and $\delta_{S}$ denote the energy consumption for single failed and successful RA event, respectively. Note that $\delta_{F}$ and $\delta_{S}$ are random variables because $P_{3}$ is the random variable that is determined by the trial number and the path-loss of device as in Equations (5) and (7). Therefore, $P_{3}$ can be expressed as $P_{3}\left(t,x\right)\left[mW\right]=P\left(r\right)P\left(x\right)P_{c}$, where P(r) is the ramping component; $r=min(t,K_{max}\left(x\right))$, P(x) is the path-loss component, and $P_{c}$ is the target power, which is a constant value. From the previously-described power consumption model, $\delta_{F}$ and $\delta_{S}$ can be simply expressed as follows:

(26) $$\begin{array}{c} \delta_{F}=\theta_{1}P_{3}\left(t,x\right)+\theta_{2},\\ \delta_{S}=\theta_{3}P_{3}\left(t,x\right)+\theta_{4}, \end{array}$$

where $\theta_{1},\theta_{2},\theta_{3}$, and $\theta_{4}$ are constants defined by the consumption model. In the previous section, because the analysis was performed based on $Z_{u}=k$ rather than $X_{u}=x$, Equation (26) can be reformulated as follows:

(27) $$\begin{array}{c} {\bar{\delta }}_{F,k}\left(t\right)=\theta_{1}{\bar{P}}_{3,k}\left(t\right)+\theta_{2},\\ {\bar{\delta }}_{S,k}\left(t\right)=\theta_{3}{\bar{P}}_{3,k}\left(t\right)+\theta_{4}, \end{array}$$

where ${\bar{\delta }}_{F,k}\left(t\right)$ and ${\bar{\delta }}_{S,k}\left(t\right)$ denote the average energy consumption of device, which is located within zonek with the trial number *t*, having single failed and successful RA events, respectively. Here, ${\bar{P}}_{3,k}\left(t\right)$ can be calculated using the average path-loss of the devices within zonek. Therefore, ${\bar{P}}_{3,k}\left(t\right)=P\left(r\right)\bar{P}\left(k\right)P_{c},$ where P(r) is the ramping component where r=min(t,k), $\bar{P}\left(k\right)$ is the average path-loss of devices in zonek.

The energy consumption of a device located within zonek that succeeds in acquiring a connection after a number *t* of RA trials is denoted by $w_{S}\left(t,k\right)$. Moreover, $w_{F}\left(k\right)$ denotes the energy consumption of a device located within zonek that fails to acquire a connection. Then, $w_{S}\left(t,k\right)$ and $w_{F}\left(k\right)$ can be derived as follows:

(28) $$\begin{array}{cc} w_{S}\left(t,k\right) & =\sum_{t=1}^{K_{t,k}-1}{\bar{\delta }}_{F,k}\left(t\right)+\left(t-K_{t,k}\right){\bar{\delta }}_{F,k}\left(K_{t,k}\right)+{\bar{\delta }}_{S,k}\left(K_{t,k}\right),\\ w_{F}\left(k\right) & =\sum_{t=1}^{K_{t,k}}{\bar{\delta }}_{F,k}\left(t\right)+\left(N_{max}-K_{t,k}\right){\bar{\delta }}_{F,k}\left(K_{t,k}\right), \end{array}$$

where $K_{t,k}=min\left(t,k\right)$, which denotes the maximum ramping number of the device with $T_{u}=t$ and $Z_{u}=k$ as defined in Equation (7). Then, the average energy consumption of a device located within zonek when the total arrival is *N*, i.e., $E[W|N,Z_{u}=k]$, is calculated using Equations (23) and (24) as follows:

(29) $$E\left[W|N,Z_{u}=k\right]=\sum_{t=1}^{N_{max}}w_{S}\left(t,k\right)P_{CS,T=t|n,Z=k}+w_{F}\left(k\right)P_{CF|N,Z=k}.$$

Finally, E[W|N=N] is

(30) $$E\left[W|N\right]=\sum_{k=1}^{N_{max}}E\left[W|N,Z_{u}=k\right]P\left(Z_{u}=k\right).$$

## 5. Simulation Results

In this section, the simulation results are provided to show the performance of the proposed Msg3 PR scheme. A discrete event simulator is built to analyze the performance of the RA procedure and the effect of the capture. The simulator implements the RA procedure defined in Section 2.1, and also implements the capture effects by adjusting the capture condition as Equation (1). Table 2 summarizes the commonly used simulation parameters in Section 5.1 and Section 5.2.

Through the analysis results defined in Section 4 and the simulation results, the steady-state performances in terms of the RA throughput, the connection failure probability and the average energy consumption are presented in Section 5.1. In Section 5.2, the possibility of coexistence of the Msg3 PR scheme with other typical RA-related schemes is presented by showing simulation results.

### 5.1. Performance of the Msg3 PR Scheme

In this subsection, we present the analysis (Anal.) and simulation (Sim.) results of the Msg3 PR scheme when the network is in a steady-state. Because the steady-state analysis model defined in Section 4 can be obtained when *N* is determined, the simulations also performed with fixed *N*. Therefore, we generate static new arrival at each RACH to converge the total arrival to *N*. Moreover, the locations of newly arrived devices are determined by the uniform distribution.

The simulation model assumes a steady-state condition. In other words, the number of arrivals and the distribution of the trial and the ramping number of devices converge.

We show RA throughput performance in terms of PRS, $N_{max}$, and the number of preambles in an RACH, *V*. Furthermore, the connection failure probability analysis and the average energy consumption analysis according to PRS change are additionally performed to further analyze the performance of Msg3 PR scheme. The performance metrics used in the study are defined as follows.

The RA throughput performance is evaluated in terms of PRS, $N_{max}$, and the number of preambles in a RACH, *V*. Furthermore, analyses of the connection failure probability and the average energy consumption according to PRS change are additionally performed to further analyze the performance of the Msg3 PR scheme. Note that we use the same parameters relative to energy consumption model as in Table 2 in the simulation. The performance metrics used in the study are defined as follows:

- RA throughput
  (Anal.)—Equation (18).
  (Sim.)
  $$E\left[S|N\right]=\frac{Total\;\#\;ofRA\;Success\;Devices}{N\times SimNumber},$$
- Connection failure probability
  (Anal.)—Equation (25).
  (Sim.)
  $$P_{CF|N}=\frac{Total\;\#\;of\;Connection\;Failed\;Devices}{Total\;\#\;of\;RA\;Completed\;Devices},$$
- Average energy consumption
  (Anal.)—Equation (30).
  (Sim.)
  $$E\left[W|N\right]=\frac{Total\;E.C\;of\;RA\;Completed\;Devices}{Total\;\#\;of\;RA\;Completed\;Devices}.$$

In each figure, the analysis results are displayed as lines, and the simulation results are displayed as markings if we performed both an analysis and a simulation.

#### 5.1.1. The Consequences of the Capture Effect and Its Properties

Before discussing the performance metrics of the Msg3 PR scheme, we describe the consequences of the capture effect and its properties. Table 3 shows the RA success probability for given ramping numbers defined as in Equation (20), when the number of accesses is high, i.e., $P(S_{u}|N=60,R_{u}=r)$. As seen in Table 3, $P(S_{u}|N=60,R_{u}=r)$ increases as *r* increases. For example, $P(S_{u}|N=60,R_{u}=1)=4.9\%$, whereas $P(S_{u}|N=60,R_{u}=8)=28.4\%$ when PRS=1 dB. In addition, $P(S_{u}|N=60,R_{u}=1)=4.9\%$, whereas $P(S_{u}|N=60,R_{u}=8)=99.9\%$ when PRS=4 dB. In other words, the probability of RA success for a device with $R_{u}=8$ is 5.7 times that of a device with $R_{u}=1$ when PRS=1 dB. Furthermore, in the case of PRS=4 dB, the difference in the success probability is further increased to approximately 20.4 times. From Table 3, the RA success probability increases as the Msg3 PR scheme uses higher PRS values.

**Figure 5:**
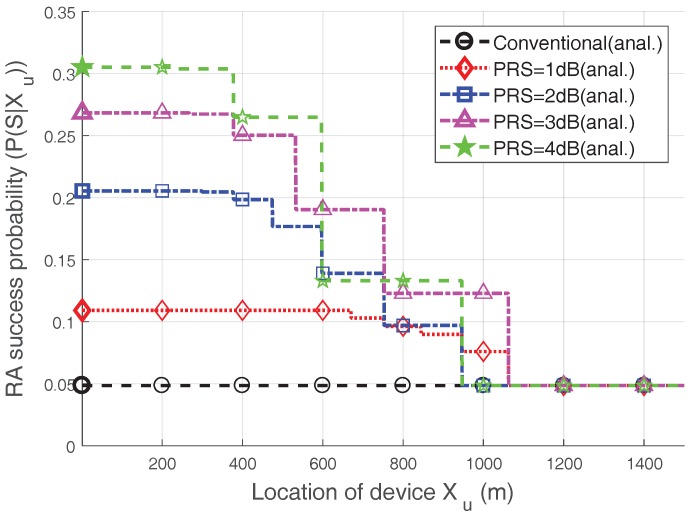
RA success probability according to the location of the device with N=60. i.e., $P(S_{u}|N=60,X_{u}=x)$.

However, this is not always true, as seen in Figure 5, which shows the RA success probability according to the location of the device. Note that the heavy-access load (N=60) case is considered, i.e., $P(S_{u}|N=60,X_{u}=x),$ which can be derived from $P(S_{u}|N=60,Z_{u}=k)$. We observe that the RA success probability decreases as the device moves away from the eNB. This is because $K_{max}\left(x\right)$ defined in Equation (6), decreases as *x* increases, and thus, the capture probability declines. One interesting point in Figure 5 is that the RA success probability is not necessarily high when the PRS is high; in other words, the trend noted in Table 3 is not always true. Based on the distance, the RA success probability when PRS=4 dB is often less than the RA success probability with PRS=3 dB. Moreover, even when *x* is between approximately 600 m and 700 m, the RA success probability for a PRS=4 dB is lower than that with a PRS=2 dB. The reason is that having too large of a PRS value in certain areas lowers the maximum ramping number, as seen in Equation (6), and, therefore, the RA success probability can be decreased. As a result, as the PRS increases, the devices close to the eNB have a higher probability of being captured, while the devices further from the eNB cannot perform power ramping well due to their lower $K_{max}$. Hence, we can make an inference about performance: an increase in PRS does not necessarily lead to an increase in RA performance.

#### 5.1.2. The RA Throughput

**Figure 6:**
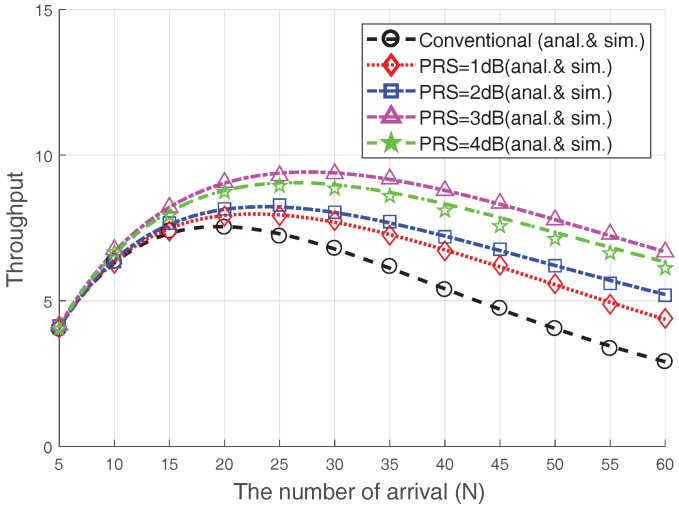
Steady-state RA throughput in terms of power ramping steo (PRS).

Figure 6 illustrates the RA throughput when the PRS is changed with V=20 and $N_{max}=8$. In addition, we compare the performance of the conventional case, which does not consider the capture effect of Msg3 (i.e., $E[S_{u},M_{v}=0|N]$) [4,5,9,14]. The RA throughput is clearly improved using the Msg3 PR scheme; particularly when heavy congestion occurs (i.e., N=3V=60), the RA throughput for PRS=1 dB, 2 dB, 3 dB, and 4 dB is improved by approximately 48%,80%,137%, and 105%, respectively, as compared to the conventional case. This is due to the fact that the capture effect may occur among the devices that suffer from collisions that reduce the RA throughput, and the Msg3 PR can increase the capture probability. Note that the increase in PRS does not necessarily lead to an increase in RA throughput, as previously mentioned. In particular, the RA throughput in the case of 4 dB is lower than that in the case of 3dB because the RA success probability of 4 dB-case is lower, as seen in Figure 5. Therefore, it is important to choose a proper PRS value. For example, choosing PRS=3 dB would be preferred in terms of the RA throughput.

**Figure 7:**
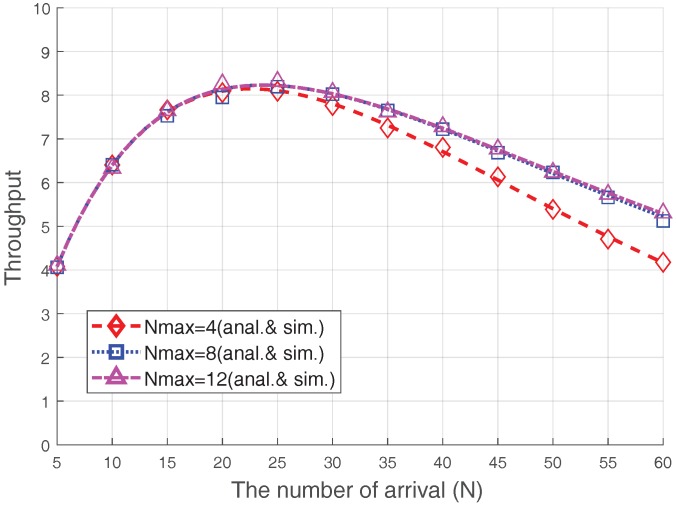
Steady-state RA throughput in terms of $N_{max}$.

Figure 7 illustrates the effect of changes in $N_{max}$ on the RA throughput when PRS=2 dB and V=20. Comparing the RA throughputs in the cases when $N_{max}=\;4$ and $N_{max}=\;8$, a throughput difference is observed when the number of accesses is large because, if the congestion level is not severe (N≤25), retransmissions are not likely to occur more than four times. Hence, there is no throughput difference between these two cases. However, if the congestion level becomes severe, the trial number of most devices will reach to $N_{max}$ due to severe collisions. Therefore, in this case, the probability of capture increases $N_{max}$ is larger. However, this observation does not apply to the $N_{max}=\;12$ case; the performance difference between the cases with $N_{max}=\;8$ and 12 is negligible. This is due to the following reasons: (1) most devices (except the devices near the eNB) could not increase their ramping number enough due to the ramping number limitation, $K_{max}$, and (2) the devices near the eNB are often captured before the ramping number is significantly increased. As a result, if $N_{max}$ becomes sufficiently large, the overall throughput is no longer affected.

**Figure 8:**
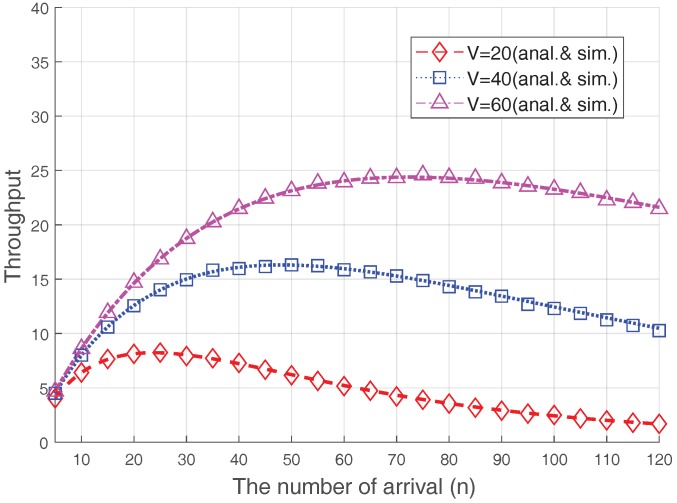
Steady-state RA throughput in terms of *V*.

In Figure 8, we also show the RA throughput varying with the size of the preamble set, *V*, where PRS=2 dB and $N_{max}=8$. As analyzed in other studies [5,6,8,12] (or, the conventional case in shown Figure 6), the RA throughput is maximized when the number of accesses is the same as the preamble size, i.e., n=V. However, if the Msg3 PR is used, the number of throughput-maximizing arrivals increases significantly. This result further demonstrates that, because of the capture effect on the RA procedure, optimization is needed if diverse RACH congestion-control schemes, such as ACB or M2M-specific back-off, are used.

#### 5.1.3. The Connection Failure Probability

**Figure 9:**
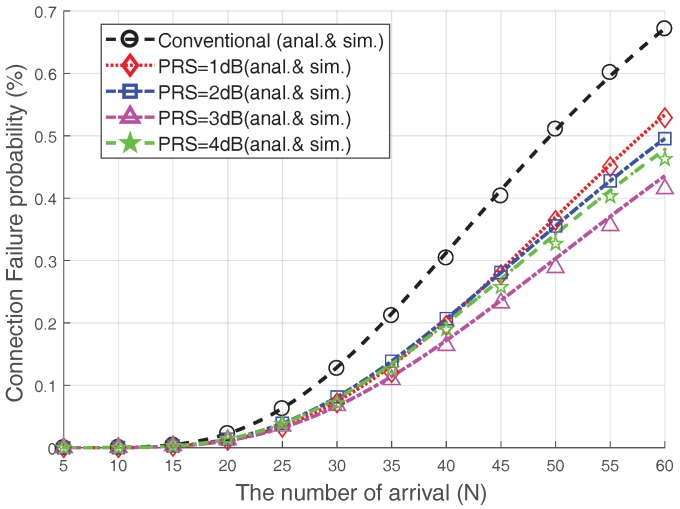
Connection failure probability ($N_{max}=8$, V=20).

Figure 9 presents the relationship between the PRS and the connection failure probability when V=20 and $N_{max}=8$. From Figure 9, we can see that, by increasing the number of accesses, the connection failure probability increases due to severe collisions. In the conventional case, almost 70% of devices experience a connection failure when the number of arrivals surges (N=60). The Msg3 PR scheme reduces the connection failure probability significantly. For example, under heavy access load (N=60), the connection failure probability could be reduced by approximately 42% when Msg3 PR with PRS=3 dB is used, as compared with the conventional case. In addition, the connection failure probability of the 3 dB-case is lower than that of the 4 dB-case.

#### 5.1.4. The Average Energy Consumption

**Figure 10:**
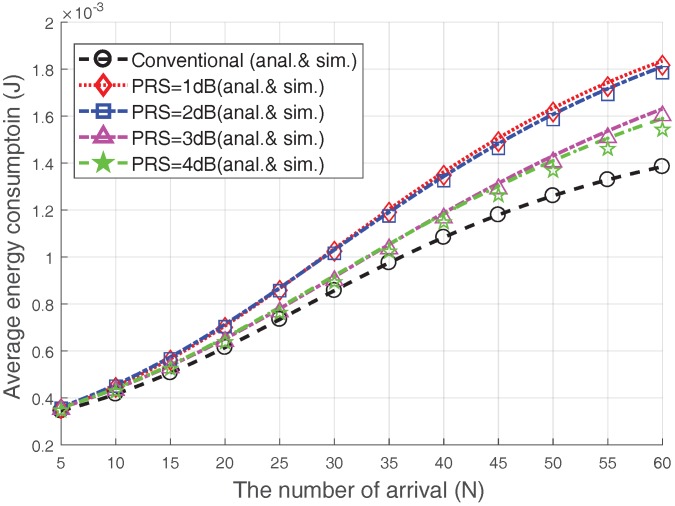
Average energy consumption of devices that completed the RA procedure ($N_{max}=8$, V=20).

Figure 10 presents the average energy consumption of the devices that completed the RA. We plot the performance when V=20 and $N_{max}=8$. The average energy consumption of the devices increases with increasing the number of accesses; this is because the RA success probability decreases due to the congestion. Since most energy consumption occurs during transmission, the Msg3 PR scheme consumes more energy than the conventioal case. However, increasing the PRS does not necessarily increase the energy consumption. Surprisingly, the Msg3 PR with PRS=3 dB or 4 dB consumes less energy than the Msg3 PR with PRS=1 dB or 2 dB. For example, the Msg3 PR consumes approximately 13% more energy than the conventional case when PRS=3 dB or 4 dB, while it consumes approximately 30% more energy than the conventional case when PRS=1 dB or PRS=2 dB. From this observation, we can argue that the energy consumption reductions due to the increase in the RA success probability are greater than the increased energy consumption due to increased transmission power results from PRS increment. In conclusion, choosing proper values of PRS and $N_{max}$ for the Msg3 PR scheme can improve the RA throughput and lower the connection failure probability with relatively little additional energy consumption.

### 5.2. Coexistence with Other Schemes

Because the Msg3 PR scheme only controls the Msg3 transmission power, it can easily coexist with other RA-related schemes. In this sub-section, we discuss the possibility of coexistence of the Msg3 PR scheme with other typical RA-related schemes through simulation results.

The simulations assume bursts of M2M traffic. In [20], the M2M traffic arrival is modeled as a beta-distribution to express the burstiness of M2M traffic. Let g(τ) denote the probability that each device is activated at time τ. Then,

(31) $$g\left(\tau \right)=\frac{\tau^{a-1}{\left(T_{A}-\tau \right)}^{b-1}}{{T_{A}}^{a+b-2}\beta \left(a,b\right)},\;0\leq \tau \leq T_{A},$$

where β(a,b) is the beta-function, $T_{A}$ is the activation time which denotes the period during which all devices are activated, and a=3,b=4 for bursty-M2M traffic. In this simulation, 10,000 devices are uniformly distributed in a cell, and each device generates traffic only once. That is, when a device experiences connection failure/success, it does not participate in the RA again. The activation time, $T_{A}$, is 10 s. Optimal ACB is assumed in this simulation, and the barring time and back-off window size are 200 ms and 20 ms, respectively.

**Figure 11:**
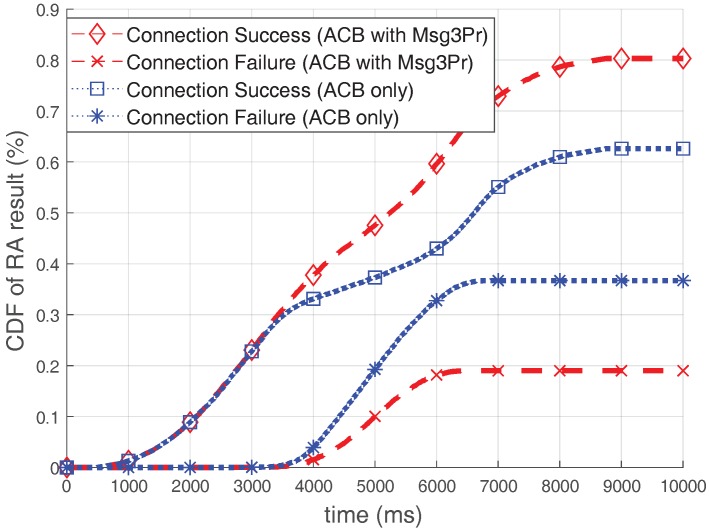
Cumulative distribution function (CDF) of RA results when optimal acess class barring (ACB) is used.

Figure 11 illustrates the cumulative distribution function (CDF) of the RA results over time when the optimal ACB is used. The ACB scheme controls the number of participants, so that the efficiency of the RACH could be maximized. Therefore, if severe congestion is predicted, it protects the RACH efficiency by blocking accesses of devices, but it does not improve RA throughput itself. As seen in Figure 11, the number of connection failed-devices is increased due to bursty arrivals if only the ACB is used. However, when using the ACB with the Msg3 PR, the number of devices experiencing connection failure is much smaller than in the case of using the ACB only. Because the Msg3 PR scheme improves the RA throughput, approximately 20% of devices (almost 2000 devices) are more successful in acquiring connections by using the Msg3 PR together. Furthermore, this performance is the result when the ACB is performed without considering the RA efficiency improvement due to the Msg3 PR scheme. Therefore, although it is not the scope of this study, a larger performance improvement can be expected if ACB optimization is performed considering Msg3 PR.

## 6. Conclusions

RACH congestion problems resulting from massive access by the M2M devices cause severe performance degradation on both the eNB and M2M devices—for example, wasting the uplink resources such as RACH and PUSCH, and increasing the connection failure probability hindering the M2M communications.

We conducted a study of the influence of the capture effect on the RA performance. It was observed that the RA performance could be improved when the Msg3 was captured by the eNB even if collision occurred. Because the capture effect could occur when the received signal powers were differentiated by the eNB, we proposed the Msg3 PR scheme that exploited the power ramping scheme to increase the capture probability.

A steady-state analysis model was provided to show the performance of the proposed scheme in terms of the RA throughput, the connection failure probability, and the average energy consumption. Numerical results of analysis and simulation results showed that the Msg3 PR scheme with proper parameters increases the RA throughput (and also increased the PUSCH efficiency), even under heavy access loads. Moreover, the proposed scheme reduced the connection failure probability, which disturbs the M2M communications, by slightly increasing the average energy consumption.

Moreover, by conducting additional simulation, we discussed the benefits of using the proposed scheme and the effects of coexistence with other RA-related technologies. The simulation results show that the proposed algorithm can be combined with other technologies to achieve a larger performance improvement. In future work, further optimization could be performed for RA-related technologies using the Msg3 PR scheme.

## Figures and Tables

**Table 1:** Notation summary.

Notation	Description
N/N	a set of devices participate RA/the size of N
$M_{v}$	the number of devices that select preamble *v* among terminals excluding device *u*
$V_{u}$	a preamble selected by device *u*
$T_{u}$	the RA trial number of device *u*
$R_{u}$	the ramping number of device *u*
$S_{u}$	the event that device *u* succeeds in the RA procedure
$P_{u}\left({P_{u}}^{rx}\right)$	transmission (received) power of (from) device *u*
$K_{max}\left(x\right)$	the maximum ramping number of a device located at a distance *x* from eNB
PRS	Power ramping step
$X_{u}$	distance from eNB to device *u*
$Z_{u}$	the zone where device *u* belongs
$N_{T=t}^{k}\left(N_{R=r}^{k}\right)$	a set of devices located within zonek with trial number *t* (with ramping number *r*)
${\bar{\delta }}_{F,k}\left(t\right)\left({\bar{\delta }}_{S,k}\left(t\right)\right)$	the average energy consumption of device located within zonek with the trial number *t*, having
	single failed (successful) RA events,
$w_{S}\left(t,k\right)\left(w_{F}\left(k\right)\right)$	the energy consumption of the device that succeeded (failed) in the connection
*W*	the energy consumption of device which is completed the RA procedure

**Table 2:** Simulation parameters.

Notation	Definition	Values
**Parameters Relative to RA Procedure**		
*V*	the number of preambles	20, 40, 60
$N_{max}$	maximum number of RA trial	4, 8, 12
$X^{cell}$	radius of single cell	1500 m
$X_{u}$	The distribution of devices	uniform
**Parameters Relative to Power Model**		
PIRTP	Preamble initial receive target power	−104 dBm
$P_{Cmax}$	Maximum transmit power	23 dBm
PRS	Power ramping step	1, 2, 3, 4 dB
delta	offset value	0 dBm
${PL}_{0}$	reference path-olss	127 dBm
$x_{0}$	reference point distance	1500 m
α	path-loss exponent	2
**Parameters Relative to Energy Consumption Model [10]**		
$\tau_{RA\_REP}$	periodicity of RACH	5 ms
$\tau_{RAR}$	processing delay	2 ms
$W_{RAR}$	RAR window size	5 ms
$\tau_{msg2},\tau_{msg4}$	requiring time for receiving Msg2/Msg4	1 ms
$W_{con\_sol}$	size of Contention-resolution timer	48 ms
$P_{1}$	power consumption when the device is waiting	−37 dBm
$P_{2}$	power consumption when the device is receiving	−22 dBm

**Table 3:** $P\left(S_{u}|N=60,R_{u}=r\right)\left(\%\right)$.

Power ramping step (PRS) $\backslash R_{u}$	1	2	3	4	5	6	7	8
1 dB	4.9	4.9	4.9	9.8	13.3	15.8	20.0	28.4
2 dB	4.9	4.9	12.0	21.3	36.6	59.1	81.8	95.7
3 dB	4.9	14.0	26.3	50.6	81.2	97.6	99.9	99.9
4 dB	4.9	15.3	45.0	82.7	98.9	99.9	99.9	99.9
